# Association Between Body Image and Physical Activity, Sociodemographic, and Morphological Variables in Adult Women

**DOI:** 10.3390/nu17030424

**Published:** 2025-01-24

**Authors:** Andrzej Knapik, Ryszard Plinta, Rafał Gnat

**Affiliations:** 1 Department of Adapted Physical Activity and Sport, Faculty of Health Sciences, Medical University of Silesia, 40-751 Katowice, Poland; aknapik@sum.edu.pl (A.K.); rplinta@sum.edu.pl (R.P.); 2 Institute of Physiotherapy and Health Sciences, Academy of Physical Education, 40-065 Katowice, Poland

**Keywords:** body image, physical activity, self-esteem

## Abstract

**Background/Objectives**: Studying non-pathological determinants of body image (BI) among adult women is essential to provide a holistic understanding of the factors that shape BI and thus to promote positive mental health, support tailored interventions, address societal impacts, and ultimately facilitate women’s healthier relationships with their bodies. The data available on this particular topic are sparse. The importance of BI for well-being prompted the authors to study its relationships with sociodemographic (age, education level, professional, marital, material status), morphological (body mass index—BMI) and behavioural (habitual physical activity) variables. **Methods**: A cross-sectional study of a cohort of 740 volunteer women was conducted with the one-time measurement of the dependent variables—individual components of BI—using the standard Body Esteem Scale (BES) questionnaire. **Results**: The BMI was a factor differentiating all domains of the BES. Among the analysed sociodemographic variables, intergroup comparisons showed significant effects of education and material status in the following BES domains: sexual attractiveness and weight concern. The physical condition domain showed differences between the categories of professional and material status. The level of physical activity differentiated respondents in the domains of weight concern and physical condition. **Conclusions**: Adult women are generally critical about their bodies. A factor that adversely affects the BES is an excessive BMI. Sociodemographic factors influence BI to a lesser degree. Physical activity shows a correlation with the BES domain of physical condition, particularly among young women.

## 1. Introduction

Body image (BI) is a multidimensional construct encompassing an individual’s perceptions of and attitudes towards their own body, including its physical appearance and functional aspects [1,2]. In simpler terms, it refers to “the picture we have in our minds of the size, shape, and form of our bodies” [3]. Body image can change over time, influenced by various factors, such as individual, biological, social, cultural, and historical elements, which exert differing levels of impact at different periods. The diversity of influences on BI has led to the development of various conceptualisations of the term. However, the synthesis of these approaches suggests that BI’s multidimensional nature encompasses perceptual, affective, and behavioural dimensions [4,5].

Body image also encompasses attitudes and interactions with others. A common stereotype links physical attractiveness with positive personal traits, a perception not limited to Western culture but prevalent worldwide [6]. Researchers adopting a sociocultural approach suggest that cultural values shape the perception of others according to prevailing standards of attractiveness and associated expectations. This perception of others, in turn, influences how individuals view themselves [7].

Body image can take on either a positive or negative nature. Positive BI involves an individual valuing their uniqueness, showing affection for their body, and respecting it [8,9]. Such individuals also accept aspects of their appearance that do not conform to socially imposed standards [10,11,12]. Body acceptance is strongly linked to overall self-esteem [13,14,15]. From an interpersonal perspective, empirical studies indicate that individuals with positive BI tend to associate with people who accept their bodies and exhibit a positive attitude towards them [16]. Conversely, negative BI is associated with psychological distress [17]. Its documented consequences extend beyond a diminished perception of physical attractiveness [18] to include low overall self-esteem [19], symptoms of depression [20], sexual dysfunction [21], and eating disorders [19,22,23]. It is understood that the development of negative BI results from the interplay between individual personality traits and sociocultural influences [17,24].

In modern times, the media exerts significant pressure in terms of shaping BI, often promoting slender figures. This can create challenges for individuals who perceive themselves as differing from these ideals [25,26,27,28,29,30]. As BI is a key element of self-identity, feelings about one’s body can profoundly influence how individuals perceive themselves and their capabilities [31]. It has a notable impact on self-esteem, quality of life, and overall well-being [32,33,34].

The literature review highlights a predominant focus among researchers on the negative aspects of BI, often linked to various diseases, disorders, or adverse social conditions. In contrast, the positive dimensions of BI and its associations with non-pathological factors are explored less frequently, despite their significant relevance to women’s mental, emotional, and physical well-being. Women with positive BI are more likely to experience higher self-esteem, reduced anxiety, and greater overall life satisfaction. Additionally, positive BI fosters resilience against societal pressures, unrealistic beauty standards, and age-related physical changes, enabling women to embrace their individuality and feel confident across different areas of life [8,10,11,12,13,14]. Given the importance of BI and its complex relationships with well-being, general functioning, and quality of life, it is vital to examine BI in relation to sociodemographic (e.g., age, education level, professional, marital, and material status), morphological (e.g., body mass index—BMI), and behavioural (e.g., physical activity) variables.

## 2. Materials and Methods

### 2.1. Design

**Figure 1:**
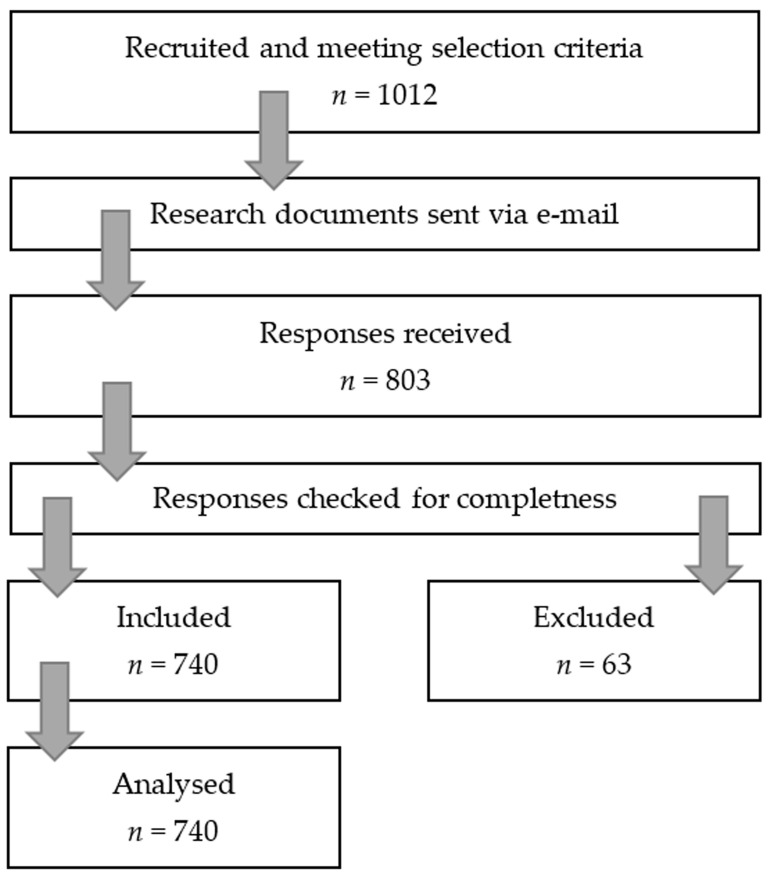
Flowchart depicting the consecutive stages of the procedure.

This was a cross-sectional study, categorising participants according to their habitual physical activity, body mass index (BMI), age, educational level, and professional, marital, and material status. The cohort consisted of 740 volunteer women. Dependent variables—individual components of BI, namely sexual attractiveness, weight concern, and physical condition—were assessed through one-time measurement using the standardised Body Esteem Scale (BES) questionnaire (Figure 1).

### 2.2. Participants

A total of 1012 women were recruited for the study, and 740 completed questionnaires (73%) meeting the completeness criteria were collected. Participants were recruited from the Silesian Voivodship in Southern Poland. The study employed a convenience sampling method: trained recruiters provided information to individuals within their networks—family, friends, student groups, colleagues, and members of fitness or sports clubs—and requested their email addresses. Links to the research questionnaires were subsequently sent to these addresses. This approach ensured voluntary and anonymous participation. Due to the planned sample size, the only established selection criteria were gender and age. The authors were solely interested in women’s BI. The lower age limit of 18 years was set in compliance with legal requirements for adulthood, while the upper limit of 59 years was chosen as it precedes the WHO-defined threshold for old age (60 years). Other variables, such as eating disorders, menstrual cycle phases, comorbid conditions, etc., were not considered, as they were not related to the stated aim of the study. It was assumed that, given the large sample size, their impact would be significantly limited. Basic demographic data are summarised in Table 1. All procedures were conducted in accordance with the Declaration of Helsinki as revised in 2013.

### 2.3. Method

This study utilised a questionnaire comprising a demographic section and standardised scales. In the demographic section, participants provided their age, height, and weight, which were used to calculate their BMI. Additional sociodemographic data were collected, including the education level (basic professional, high school, university), professional status (student, employed, unemployed), marital status (single, in a relationship), and self-assessed material status (low, average, high). These factors were treated as independent variables.

The questionnaire section included the Body Esteem Scale (BES) [35] to assess BI-related self-esteem (the dependent variable) and the Subjective Experience of Work Load (SEWL) [36,37] to evaluate physical activity (PA) levels. The BES consists of 35 items representing various body parts and functions. Participants rate their feelings towards each item on a scale of 1 to 5, where 1 indicates “definitely negative”, 2 “moderately negative”, 3 “neutral”, 4 “moderately positive”, and 5 “definitely positive”. The items are grouped into three domains: sexual attractiveness, weight concern, and physical condition. The score for each domain is calculated as the average of the ratings for its respective items, with higher scores indicating better self-esteem.

The SEWL comprises 16 items divided into three parts, including one focused on work-related items, one addressing sports, and the last addressing leisure-time PA. Points are allocated based on the physical effort associated with these activities. The remaining closed-ended statements pertain to the frequency of PA and the perception of physical exertion. The responses are scored, and calculation algorithms are applied to determine the “amount” of PA across three domains: work-related, sports, and leisure time. The combined score of these three domains represents the overall habitual PA. The SEWL outcomes were also treated as independent variables.

### 2.4. Statistical Analysis

In certain analyses, the respondents were divided into the following age groups: 18–29, 30–39, 40–49, and 50–59 years. Physical activity was also categorised: women who engaged in regular PA at least once a week, for a minimum of 1.5 h, over at least one year were classified as active; those who did not meet these criteria were classified as inactive. Body mass index categories included underweight, normal weight, overweight, and obese.

Descriptive statistics (numbers, percentages, and medians/mean values) were calculated for all variables. The internal consistency of the questionnaires was assessed using Cronbach’s alpha coefficients.

Relationships between the BES domains and other quantitative variables were examined using Pearson’s correlation coefficients. For univariate analyses, non-parametric tests were applied, including the Mann–Whitney test and Kruskal–Wallis ANOVA with a post hoc analysis. Multivariate analyses were conducted using backward stepwise regression. The critical *p*-value threshold was set at 0.05.

## 3. Results

### 3.1. Internal Consistency

The results obtained for the individual domains of the BES demonstrated strong internal consistency, with Cronbach’s alpha coefficients as follows: sexual attractiveness—0.83, weight concern—0.91, and physical condition—0.91. Similarly, the SEWL questionnaire exhibited excellent internal consistency, with a Cronbach’s alpha of 0.92.

### 3.2. Body Mass Index

**Figure 2:**
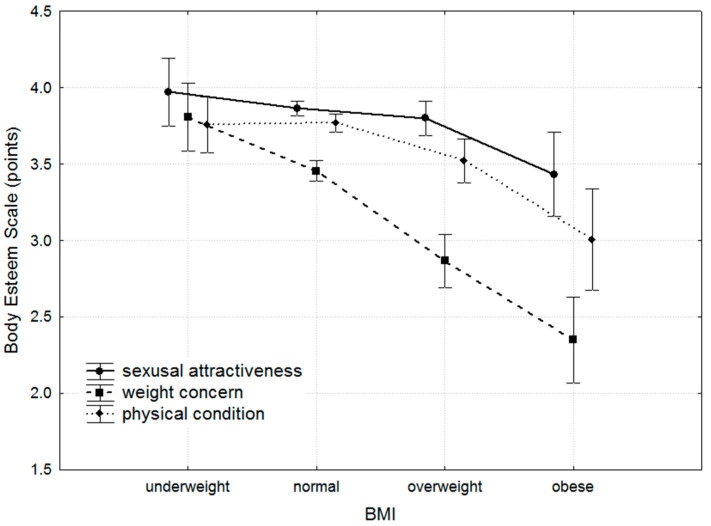
Results obtained in the Body Esteem Scale domains (mean and 95% confidence interval (whiskers)) for individual body mass index (BMI) categories in the total group of respondents.

Negative, weak-to-moderate correlations were observed between BMI and all individual BES domains. These trends were most pronounced in the 18–29 and 30–39 age groups. The strongest correlation was identified between weight concern and BMI (Table 2, Figure 2).

The performed categorisation of BMI returned a total of 51 (6.89%; mean 17.89 ± 0.48) underweight, 572 (77.30%; mean 21.48 ± 1.65) normal, 91 (12.30%; mean 26.99 ± 1.45) overweight, and 26 (3.51%; mean 34.33 ± 5.55) obese women (Kruskal–Wallis ANOVA and all post hoc comparisons *p* < 0.001).

The analysis of the BMI differences (Kruskal–Wallis ANOVA) showed that BMI was a factor differentiating BES in all domains: sexual attractiveness (*p* < 0.01), weight concern (*p* < 0.0001), and physical condition (*p* < 0.0001). Post hoc comparisons showed a number of differences (Table 3, Figure 2).

### 3.3. Sociodemographic Variables

Intergroup comparisons of the analysed sociodemographic variables revealed that education and material status significantly influenced the sexual attractiveness domain of the BES. Differences in the weight concern domain were observed only between the 18–29 and 30–39 age groups. Meanwhile, the physical condition domain showed differences between categories of professional status as well as material status (Table 4).

### 3.4. Physical Activity

The correlations between the individual BES domains and the SEWL questionnaire outcomes were weak to moderate, positive, and occasionally significant (Table 5). These correlations were particularly evident in the overall participant group, the 18–29 age category, and within the physical condition domain of the BES.

**Figure 3:**
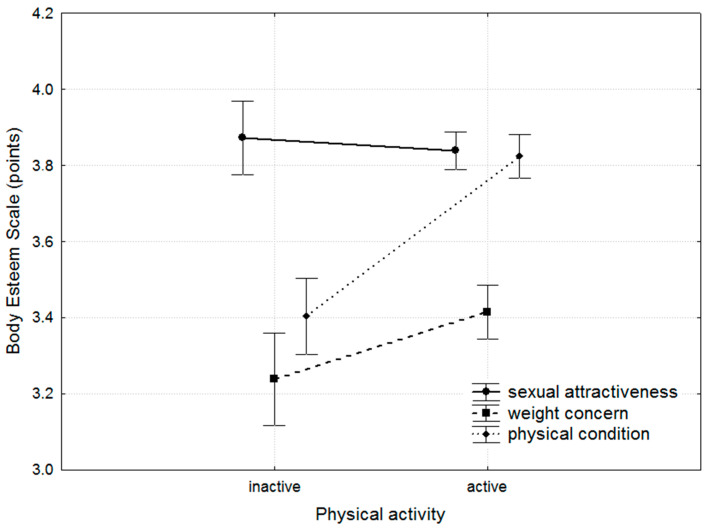
Results obtained in the Body Esteem Scale domains (mean and 95% confidence interval (whiskers)) for individual physical activity categories in the total group of respondents.

The SEWL-score-based categorisation of PA identified 538 active women (72.70%; mean score 9.22 ± 1.80) and 202 inactive women (27.30%; mean score 7.99 ± 1.92) (Mann–Whitney test, *p* < 0.001). Comparing the participants based on the defined activity criterion revealed differences in the weight concern and physical condition domains of the BES. These differences were evident in the total participant group and within the 30–39 age subgroup. In the 18–29 age subgroup, differences were observed solely in the physical condition domain (Table 6, Figure 3).

### 3.5. Regression Analysis

**Figure 4:**
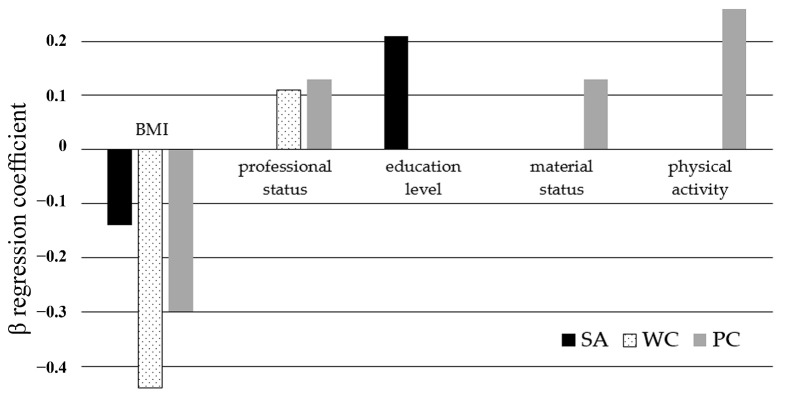
Summary of the identified significant predictors of the outcomes in the individual Body Esteem Scale domains: sexual attractiveness (SA), weight concern (WC), and physical condition (PC). Presented are β-coefficients of backward stepwise regression. Non-significant factors were omitted. BMI—body mass index.

The backward stepwise regression analysis identified several significant predictors of the BES score. Body mass index emerged as a significant predictor across all BES domains: sexual attractiveness (β = −0.14; *p* < 0.001), weight concern (β = −0.44; *p* < 0.0001), and physical condition (β = −0.30; *p* < 0.0001). As reflected by the regression β-coefficient, BMI was consistently negatively associated with the scores in all three domains. Conversely, all other identified predictors exhibited positive correlations with the BES scores. Professional status was a significant predictor in the weight concern (β = 0.11; *p* < 0.05) and physical condition (β = 0.13; *p* < 0.001) domains. Education level was found to be significant in the sexual attractiveness domain (β = 0.12; *p* < 0.01). Additionally, within the physical condition domain, material status (β = 0.09; *p* < 0.05) and PA (β = 0.26; *p* < 0.0001) were identified as significant predictors (Figure 4).

## 4. Discussion

The analysis of the results highlights a rather critical self-assessment of BI within the participant group. The medians (Table 3) and means (Figure 2) ranged between intermediate and moderately positive. On one hand, this suggests a realistic approach to the self-evaluation of BI; on the other, it may reflect a perceived gap between one’s current state and personal expectations. This phenomenon is characteristic of highly developed socioeconomic environments, where physical appearance is highly valued, and the female ideal demands continuous effort towards maintaining and improving one’s body [38,39,40].

No significant differences in sexual attractiveness were observed between active and inactive women (Table 6). This phenomenon may be explained by historical and social factors, particularly concerning traditional gender roles [41]. For men, the functional aspects of the body, such as physical fitness and activity, have historically played a far greater role in determining attractiveness [42]. Researchers interpret this as reflecting men’s long-standing role as protectors, where physical fitness and activity were essential in ensuring safety. Consequently, an active and fit man was perceived as a more desirable partner, and, even today, these traits remain key motivators for male PA [43,44]. Historically, women’s roles in most cultures were markedly different. Indicators of sexual attractiveness were, and still often are, linked to body regions perceived as crucial for reproduction, such as the breasts, abdomen, pelvic area, and face [45,46]. Features like the waist-to-hip ratio and breast size are particularly significant in determining reproductive attractiveness in women [47]. Unfortunately, anthropometric data related to these factors were not collected in this study. However, it can be speculated that women with an underweight or normal BMI may have had more favourable proportions in these areas, as a higher BMI is often associated with abdominal obesity [48]. This interpretation, of course, applies only to one specific aspect of sexual attractiveness. The significant differences in sexual attractiveness observed between the BMI categories lend further support to this argument (Table 3, Figure 2).

An intriguing finding is the absence of a statistically significant correlation between age and sexual attractiveness (*p* > 0.05). Previous studies suggest that attributes linked to fertility, such as body shape, facial features, and voice pitch, significantly influence a woman’s perceived attractiveness [49]. While the medians for sexual attractiveness were marginally higher in age groups I and II compared to groups III and IV, the hypothesis that age negatively impacts sexual attractiveness was not supported by this study. This aligns with the notion that physical attractiveness and sexual attractiveness, the latter being more biologically rooted, are distinct constructs [50]. This perspective is somewhat consistent with the observation that critical BI evaluations peak during the perimenopausal period but remain relatively stable during the pre- and postmenopausal stages [51]. Such patterns may reflect both the increased lifespan of modern humans—extending all individual life stages—and the growing influence of non-biological factors, such as evolving social and moral norms. Sexual activity, no longer solely tied to procreation, has become a recognised element of contemporary quality of life [52]. This is further supported by the observed effects of education and material status on sexual attractiveness (Table 3 and regression analysis).

In contemporary society, weight concern has become a significant component of BI. Self-perception is shaped by numerous factors, including emotional, social, cultural, and interpersonal influences. Increasingly, the media play a pivotal role in this process by promoting the ideal of the “perfect body” [53]. A slim figure is often associated with success, health, and sexual attractiveness [54]. From a public health perspective, this trend has both positive and negative implications. On the positive side, weight concern may contribute to mitigating the global obesity epidemic to some extent [55]. However, among young women and girls with a healthy BMI, it can also lead to risky behaviours, such as excessively restrictive diets, overly intense exercise routines, or even the use of weight loss drugs [56]. The prevailing “cult of thinness” is evident across all three domains of the BES (Figure 2). While the decline in BES mean values varies with increasing BMI, this trend is particularly pronounced in the weight concern domain.

When considering PA, differences in weight concern between active and inactive women were observed only in the second age group, i.e., women in their thirties. It appears that, during this stage of life, women regard PA as the most effective method of weight control. Comparisons of the weight concern scores across the categories of education level, professional status, marital status, and material status revealed no significant differences. Notably, among the three BES domains analysed, weight concern was the most critically assessed, reflecting, in many cases, an excessive focus on body weight. This may be a consequence of the previously mentioned “cult of thinness”. Research suggests that women are generally more self-critical of their body weight than men [57]. However, this observation does not hold true across all ethnic groups and cultural settings [58,59]. The regression analysis identified professional status as a partial predictor of weight concern. This finding may be linked to the professional roles and societal expectations of women in Western cultures, although further research is needed to explore this relationship in greater depth.

The results indicate that the physical condition of underweight and normal-weight women is nearly equivalent. However, as the BMI increases, the physical condition declines sharply, reaffirming the well-established fact that excess body weight negatively impacts functional capabilities. When considering PA, differences between active and inactive women were evident across the entire group of respondents. However, when age was factored in, the impact of activity on physical condition was more pronounced in younger women. This suggests that the importance placed on physical condition diminishes with age. Nevertheless, given the ongoing dynamics of social and cultural changes, this issue may require further attention. Previous studies highlight that PA is a positive factor in shaping and enhancing BI [60].

The physical condition domain of the BES revealed significant differences between students and working women. This may be explained by the temporary pressures that young women face during their studies, which can restrict their ability to maintain an active lifestyle, thereby negatively impacting their physical condition. A notable difference was also observed between women reporting an average and high material status. However, the relationship between material status and BI remains ambiguous in the context of other studies. Some reports suggest that individuals with lower financial status tend to be dissatisfied with their body shape [61], while others argue that dissatisfaction with BI is more prevalent among those with higher socioeconomic status [62]. This discrepancy is likely influenced by cultural variations.

The data presented offer a deeper understanding of the role of various non-pathological factors in shaping women’s self-perception of their bodies. From a broader perspective, this knowledge is essential in developing targeted health promotion strategies that encourage positive BI and, in turn, enhance public mental health outcomes. The findings also highlight the need to expand future research to explore the intricate interplay between pathological and non-pathological influences on BI. Investigating more specific populations could provide valuable insights. For example, women with conditions that affect body image, or adolescents, who are particularly susceptible to social pressures during their formative years, often face lasting mental health implications. By focusing on these groups, researchers can gain a more nuanced understanding of the complex interrelations among societal, psychological, and biological factors, thereby paving the way for tailored interventions that address diverse and specific needs.

The limitations of the study include reduced generalisability, as local social, economic, cultural, and religious factors, typical of a Central European population with a culture similar to that of Western Europe—predominantly Christian and politically leaning towards conservatism and centre-right ideologies—may have influenced BI and should be taken into account. Additionally, the number of respondents across the different age, BMI, and PA categories was largely uneven, which may have affected the statistical analysis. However, our primary objective was to gather a large cohort of participants, and all categorisations were performed retrospectively, making them of secondary importance compared to the main goal. Due to this same reason, such variables as eating disorders, menstrual cycle phases, comorbid conditions, etc., were not considered as selection criteria when categorising the variables. According to the authors, these important issues should be the subject of separate research using appropriate tools for that purpose. However, it was assumed that BI remains relatively stable in cases of chronic psychological disorders and that the Body Esteem Scale demonstrates a certain resilience to temporary changes in body image associated, for example, with menstrual cycle phases.

Readers should also note the use of subjective measurement tools. Nevertheless, the typical limitations associated with subjectivity were mitigated through the implementation of an anonymous approach and the use of standardised questionnaires specifically designed to address the challenges of subjective self-assessment. It is also important to note the limitations of the cross-sectional design, which does not allow for the formulation of cause-and-effect relationships.

## 5. Conclusions

Adult women tend to be highly critical of their bodies. An excessive BMI appears to be a key factor negatively associated with all three domains of the BES. Sexual attractiveness is linked to the level of education but shows no significant relationship with age or PA levels. Professional status is moderately related to BI and self-esteem in the domain of physical condition, while PA shows a correlation with physical condition, particularly among young women.

## Figures and Tables

**Table 1:** Demographic characteristics in the total group of participants.

Characteristic	Mean ± Std. Dev. (Min–Max)
Age (years)	27.73 ± 8.66 (19–59)
Body height (m)	1.67 ± 6.45 (150–197)
Body mass (kg)	62.69 ± 10.58 (42–128)
Body mass index (kg/m^2^)	22.31 ± 3.38 (16.18–41.86)

**Table 2:** Correlations between Body Esteem Scale domains and body mass index. Age groups (years) are also considered.

Variable	Age Group								Total	
	18–29		30–39		40–49		50–59			
	*r*	*p*	*r*	*p*	*r*	*p*	*r*	*p*	*r*	*p*
SA	−0.02	ns	−0.34	***	−0.05	ns	−0.12	ns	−0.12	**
WC	−0.42	***	−0.54	***	−0.30	*	−0.26	ns	−0.42	***
PC	−0.14	**	−0.37	***	−0.50	***	−0.33	ns	−0.24	***

SA—sexual attractiveness; WC—weight concern; PC—physical condition; r—Pearson’s correlation coefficient; ns—non-significant; * *p* < 0.05; ** *p* < 0.01; *** *p* < 0.001.

**Table 3:** Body Esteem Scale and body mass index categories. Age groups (years) are also considered.

Variable	BMI	Age Group								Total	
		18–29		30–39		40–49		50–59			
		Me	*p*	Me	*p*	Me	*p*	Me	*p*	Me	*p*
SA	a: U	3.85		3.54		4.62		3.69		3.85	
	b: N	3.92		4.08		3.62		3.85		3.92	
	c: OV	3.92		3.85		3.58		3.58		3.77	a–d *
	d: OB	3.69	ns	3.27	b–d **	3.73	ns	2.69	ns	3.62	b–d **
WC			a–b *								a–b *
	a: U	4.00	a–c **	3.60		4.30		3.40		4.00	a–c ***
	b: N	3.50	a–d ***	3.40	a–d *	3.60		3.50		3.50	a–d ***
	c: OV	2.50	b–c ***	2.90	b–c *	2.85		3.60		2.80	b–c ***
	d: OB	2.20	b–d *	2.00	b–d ***	3.05	ns	2.30	ns	2.30	b–d ***
PC	a: U	3.67		4.50		4.33		4.22		3.78	
	b: N	3.89		4.00		4.00		3.78		3.89	a–d ***
	c: OV	3.50		3.44		3.39		3.89		3.56	b–c **
	d: OB	3.06	ns	2.83	b–d *	3.50	ns	2.83	ns	3.17	b–d ***

BMI—body mass index; U—underweight; N—normal; OV—overweight; OB—obese; SA—sexual attractiveness; WC—weight concern; PC—physical condition; Me—median; ns—non-significant; * *p* < 0.05; ** *p* < 0.01; *** *p* < 0.001 (post hoc for Kruskal–Wallis ANOVA).

**Table 4:** Body Esteem Scale domains and sociodemographic variables.

Variable	Category	*n* (%)	SA		WC		PC	
			Me	*p*	Me	*p*	Me	*p*
Education Level (*n* = 740)	a: basic professional	62 (8.38	3.54	a–b *** a–c ***	3.30	ns	3.89	ns
	b: high school	220 (29.73)	3.92		3.50		3.67	
	c: university	458 (61.89)	3.92		3.40		3.78	
Professional Status (*n* = 740)	d: student	345 (46.62)	3.85	ns	3.40	ns	3.67	d–e *
	e: working	296 (40.00)	3.85		3.50		3.89	
	f: not working	99 (13.38)	3.77		3.40		3.88	
Marital Status (*n* = 656)	g: single	174 (26.52)	3.77	ns	3.30	ns	3.78	ns
	h: in relationship	482 (73.48)	3.92		3.40		3.67	
Material Status (*n* = 581)	i: low	160 (27.54)	3.96	i–j **	3.50	ns	3.89	j–k *
	j: average	223 (38.38)	3.77		3.40		3.67	
	k: high	198 (34.08)	3.92		3.60		3.89	
Age (years) (*n* = 740)	l: 18–29	551 (74.46)	3.92		3.50		3.78	
	m: 30–39	106 (14.32)	3.85		3.10		3.67	
	n: 40–49	50 (6.76)	3.62		3.40		3.67	
	o: 50–59	33 (4.46)	3.69	ns	3.50	a–b *	3.78	ns

SA—sexual attractiveness; WC—weight concern; PC—physical condition; Me—median; ns—non-significant; * *p* < 0.05; ** *p* < 0.01; *** *p* < 0.001 (marital status—Mann–Whitney test, other variables—post hoc for Kruskal–Wallis ANOVA).

**Table 5:** Body Esteem Scale and physical activity (SEWL total score). Age groups (years) are also considered.

Variable	Age Group								Total	
	18–29		30–39		40–49		50–59			
	*r*	*p*	*r*	*p*	*r*	*p*	*r*	*p*	*r*	*p*
SA	0.13	**	0.02	ns	0.16	ns	0.35	*	0.13	**
WC	0.12	*	0.09	ns	0.20	ns	0.16	ns	0.13	**
PC	0.43	***	0.32	**	0.34	*	0.32	ns	0.40	***

SA—sexual attractiveness; WC—weight concern; PC—physical condition; r—Pearson’s correlation coefficient; ns—non-significant; * *p* < 0.05; ** *p* < 0.01; *** *p* < 0.001.

**Table 6:** Body Esteem Scale and Subjective Experience of Work Load scale categories. Age groups (years) are also considered.

Variable	Activity	Age Group								Total	
		18–29		30–39		40–49		50–59			
		Me	*p*	Me	*p*	Me	*p*	Me	*p*	Me	*p*
SA	A	3.85	ns	3.77	ns	3.62	ns	3.65	ns	3.85	ns
	IA	3.92		4.00		3.62		3.69		3.92	
WC	A	3.50	ns	3.10	*	3.40	ns	3.50	ns	3.50	*
	IA	3.40		3.00		3.90		3.40		3.30	
PC	A	3.89	***	3.89	**	3.67	ns	3.89	ns	3.89	***
	IA	3.33		3.33		3.67		3.33		3.33	

A—active women; IA—inactive women; SA—sexual attractiveness; WC—weight concern; PC—physical condition; Me—median; ns—non-significant; * *p* < 0.05; ** *p* < 0.01; *** *p* < 0.001 (Mann–Whitney test).

## Data Availability

The data presented in this study are available on request from the corresponding author due to legal reasons.

## Abbreviations

The following abbreviations are used in this manuscript:

BI	Body Image
BMI	Body Mass Index
BES	Body Esteem Scale
SEWL	Subjective Experience of Work Load
PA	Physical Activity
